# Indigenous farming methods and crop management practices used by local farmers in Madibeng local municipality, South Africa

**DOI:** 10.1038/s41598-025-91210-w

**Published:** 2025-03-14

**Authors:** Thembeni A. Khumalo, Mompati V. Chakale, John A. Asong, Adeyemi O. Aremu, Stephen O. Amoo

**Affiliations:** 1 Indigenous Knowledge Systems Centre, Faculty of Natural and Agricultural Sciences, North-West University, Private Bag X2046, Mmabatho, 2790 South Africa; 2 Agricultural Research Council–Vegetables, Industrial and Medicinal Plants, Private Bag X293, Pretoria, 0001 South Africa; 3 Unit for Environmental Sciences and Management, Faculty of Natural and Agricultural Sciences, North-West University, Private Bag X1290, Potchefstroom, 2520 South Africa

**Keywords:** Biodiversity, Conservation, Ethnopedology, Food security, Pest management, Sustainable livelihood, Environmental sciences, Environmental social sciences

## Abstract

This study investigated indigenous farming methods, practices, and crop management strategies used by farmers in local communities within the North West Province, South Africa. A survey was conducted using semi-structured interview guide with 49 participants recruited through the snowball sampling technique. Data were analysed using ethnobotanical indices, including frequency of citation (FC), use value (UV), and relative frequency of citation (RFC). The participants identified four distinct soil types, along with different ways for soil preparation. Five soil fertilisation materials were mentioned with mixed cropping (intercropping) (95.9%) and crop rotation (93.8%) being the most used farming systems. Additionally, we identified 10 plant species belonging to seven families that were used to manage crop pests and diseases. The plant with the highest RFC was *Allium dregeanum* Kunth (RFC = 0.88) while *Tulbaghia violacea* Harv. had the second highest RFC (0.73) and the highest UV (0.10). The families with the highest number of plant species were Fabaceae (3 plants) and Alliaceae (2 plants). In terms of plant parts used, bark was the most cited (53%), followed by whole plant (32%) and fruit (10%). Overall, this study underscores the importance of indigenous crop farming methods and practices towards household food security and general well-being.

**Supplementary Information:**

The online version contains supplementary material available at 10.1038/s41598-025-91210-w.

## Introduction

Indigenous farming methods and practices are historical endeavours associated with a particular ethnic group that are often explored by local and subsistence farmers for crop production in sustainable manner, as evident from different parts of the world^1–5^. Indigenous communities utilise natural resources to drive the agricultural productivity while sustaining their household food security^6–8^. The relationship that Indigenous people have with their biophysical environment allowed them to use natural resources in a sustainable way^9–12^. This knowledge, particularly in agriculture, has been vital for survival, especially in challenging environments. In Africa, many rural communities significantly rely on indigenous knowledge for their daily survival including agricultural productivity^13^. Historically, Indigenous peoples have developed and utilised locally sourced knowledge to adapt to their natural surroundings, thereby effectively managing difficult conditions such as droughts, pests, and poor soil quality^14,15^. In response to these challenges, these communities have crafted agricultural techniques that promote resilience and sustainability in the face of harsh environments^16^. Although indigenous farming methods and practices have stood the test of time and are still being utilised in many local communities, they are overlooked and often regarded as primitive^17–19^.

Recently, there is a renewed interest in exploring indigenous farming methods and practices, especially in the face of challenges such as decline in yield due to pests and diseases^20–24^. When compared to conventional agricultural practices including the use of chemicals, indigenous knowledge is relatively affordable. Having been refined and tested over a long period, the application of indigenous knowledge remains invaluable for maintaining biodiversity, fostering cultural heritage, and providing adaptive strategies to local environmental conditions^6^.

In various African countries, local farmers often apply unique methods and practices at different stages (such as during soil preparation, soil fertilisation, planting, and crop management) of crop production for enhanced yield^24–26^. Traditional farming systems may offer cues that can be explored to overcome certain limitations in modern agriculture, maximize and secure local or household food production and biodiversity^27–29^. Additional benefits associated with these practices include the potential to promote local diets and generate income for rural households^30^. One of the predominant activities in Madibeng local municipality (South Africa) is commercial agriculture which is based on monoculture and the use of chemical fertilizers to enhance crop production^31,32^. Although the people work in the commercial farms, they still produce crops in home gardens for their households using indigenous methods and practices.

This study aims to document the indigenous farming methods and crop management practices currently utilised in Madibeng Local Municipality, and to explore their potential to enhance food security in the region. By doing so, the study is envisaged to enhance the existing body of scientific literature on indigenous agricultural practices, preventing valuable knowledge from becoming extinct. Furthermore, this research is positioned to raise awareness about the benefits and potential of traditional farming systems in sustainable agriculture, food production, ecosystems, soil health, and biodiversity. This study also aligns with the global agenda for sustainable development, particularly in relation to the United Nations Sustainable Development Goals (UN SDGs) 2 (Zero Hunger), 3 (Good Health and Well-being), and 12 (Responsible Consumption and Production).

## Materials and methods

### Description of study area

**Fig. 1:**
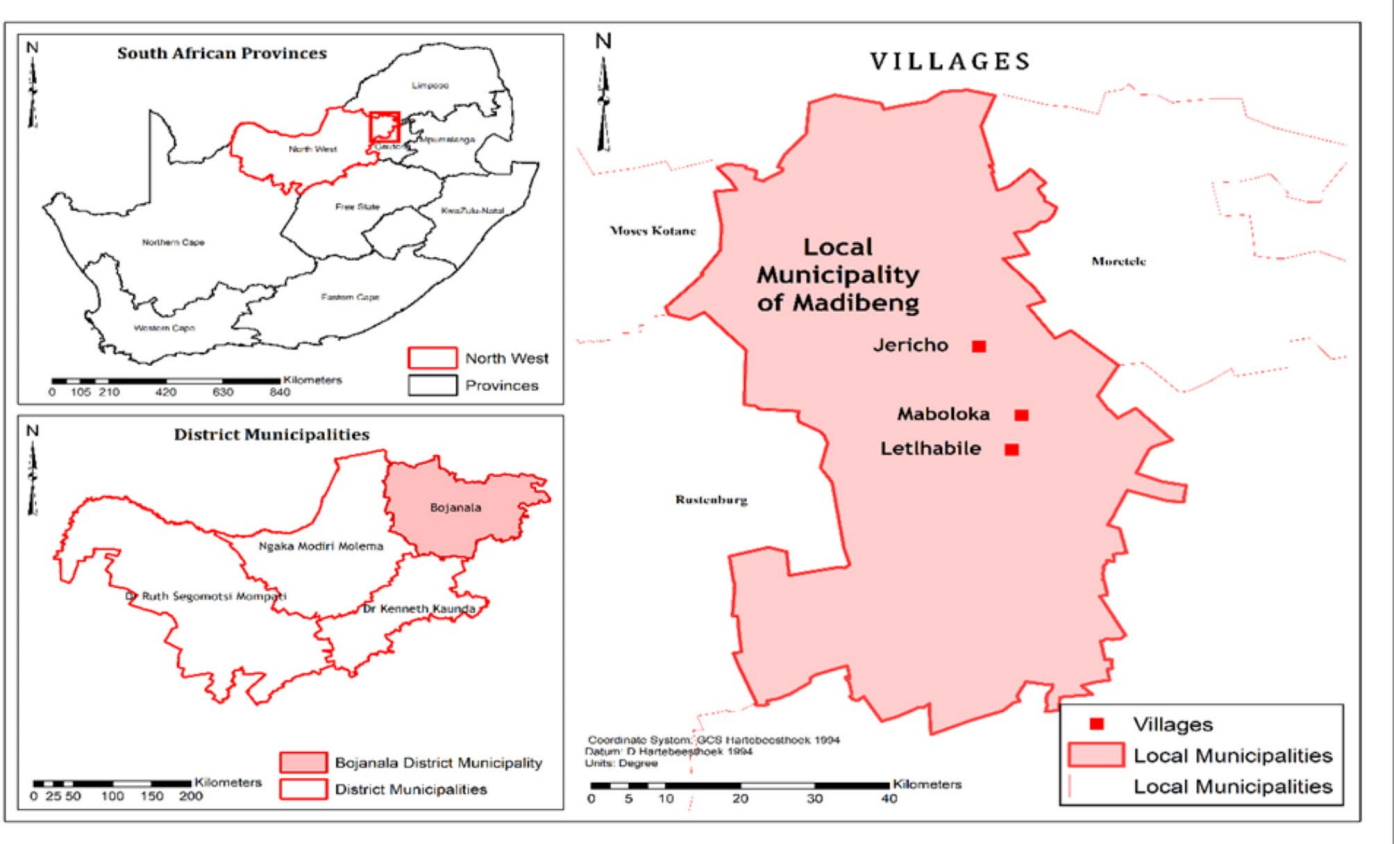
An overview of the three selected communities in Madibeng local municipality in Bojanala District, North West Province, South Africa.

The study was conducted in three selected communities within the Madibeng local municipality located in the Bojanala district municipality in the North-West Province, South Africa (Fig. 1). The selected district lies between latitude  26°34’35.2”S 22°50’57.4”E, and longitude 27°44’59.99”E of the North-West Province, covering 3,839 km^2^ in land mass^33^. Madibeng is a central area in the North West Province, with Pretoria, Johannesburg, Rustenburg and Krugersdorp as bordering cities. The municipality has 31 wards (geopolitical divisions) of which 10 fall in the urban areas and 21 in the rural areas. It includes approximately 43 communities and 900 farm areas. It is strategically located in relation to Gauteng and Limpopo provinces, and positioned along the Heritage Route, linking the World Heritage Site with the Pilanesberg and Madikwe Game Reserves^33^. The average annual rainfall is about 360 mm, mostly experienced in the summer months between October and April, while summer temperature ranges from 17 to 31 °C and winter temperature ranges from 3 to 21 °C. The farming activity is mainly subsistence mixed farming where crop and animal production dominate^34^.

Madibeng is characterised by diverse economic sectors, such as agriculture, mining, manufacturing and tourism. Based on 2022 data, the total estimated population of Madibeng local municipality was 522, 566 people. Madibeng local municipality was selected due to its high biodiversity and economic activities. Furthermore, the selection of communities was based on having preserved traditional knowledge systems that have been passed down through generations, and this was validated by those in authority of the municipality. These practices are often adapted to the specific environmental and climatic conditions of the region, making them highly relevant for sustainable agriculture. Additionally, rural communities tend to have less exposure to modern agricultural technologies, allowing for a more authentic and undiluted study of indigenous methods. In this study, rural communities were identified based on their strong agricultural heritage and the presence of knowledgeable elders and practitioners who actively use indigenous farming techniques. These communities provide a rich context for studying the intricacies of traditional practices and offer valuable insights into how these methods contribute to their resilience and sustainability. Furthermore, the population is dominated (89.3%) by Black Africans, and Setswana is the most spoken language. Major crops grown in the study areas include maize, sunflower, wheat, groundnuts. Home garden crops include potatoes (*Solanum tuberosum*), garlic (*Allium sativum* L.), cabbage (*Brassica oleracea* L.) and spinach (*Spinacia oleracea* L.). In addition, livestock and bees are major parts of the farming system^15^.

### Data collection

An ethnobotanical field survey was conducted from 4th October to 5th December 2023 across the three selected rural communities (Fig. 1). The study adopted a mixed method research approach^35^. A face-to-face session using a semi-structured interview guide prepared in English and translated to Setswana (local language) was conducted to collect data, and the data were subsequently translated to English. The semi-structured interview guide yielded insightful knowledge to develop and generate a rich understanding of the knowledge and skills related to indigenous farming methods and crop management practices^36^.

Data collection was done in two phases: the first involved interviews, and the second included field visit for plant collection, following the technique described by Alexiades and Sheldon^37^. Responses from individual interviews were cross-checked with other participants within the same communities to ensure reliability. Field walks were conducted to collect plants mentioned during interviews. Study participants were systematically sampled from the three communities based on their knowledge on indigenous crop farming methods and practices.

Given the difficulties in accessing populations that are actively involved in the application of indigenous knowledge for agricultural practices, snowball sampling was used to recruit and screen eligible participants^38–40^. The study targeted indigenous knowledge holders and local farmers, as they are the primary custodians of traditional agricultural practices within the community. These participants were selected for their in-depth knowledge and experience with indigenous farming methods, which were central to the research objectives. A total of 49 participants were involved in the study. For botanical identification, plant species were collected during the field visit, identified and deposited in the herbarium at SD Phalatse (UNWH) North-West University, Mahikeng, South Africa. Furthermore, plant names were verified using the World Flora Online (https://powo.science.kew.org/, accessed on 31 August 2024).

### Ethical considerations

Prior to data collection, ethical approval was obtained from the Faculty of Natural and Agricultural Sciences Research Ethics Committee (FNASREC) at North-West University (Ethics approval number: NWU-01409-23-A9). As the study involved human participants, we conducted the research in accordance with the declaration of Helsinki. A permit for plant collection was secured from the North-West Provincial Department of Rural, Environmental, and Agricultural Development (NW-READ) (Permit number: 49711). Permission was granted by traditional authorities to conduct the study, and all participants signed informed consent forms. Participants were approached to seek their consent to participate in the study following detailed and clear explanation on the purpose of the research. Any use of the study information beyond scientific publications requires prior consent from the Indigenous Knowledge Holders who are the participants, and their agreement on possible benefits before any commercialisation.

### Data analysis

Descriptive statistics (frequency and percentage) were used to identify and describe the socio-demographic characteristics of the participants^41^. Thematic content and ethnobotanical indices were used to analyse the data collected. Following the interviews, the data were transcribed and verified for coherence and saturation. The information from various participants was compared to each other to uncover trends and themes. The emergent themes were linked to data sections with corresponding codes such as participant socio-demographic information, frequently identified indigenous knowledge used for crop protection, and plant usage process. When no new data, codes, or themes came from the material, it was considered that saturation had been reached, hence, the relatively small sample size of 49 was used. Based on the previous ethnobotanical indices^42,43^, three quantitative parameters: frequency of citation (FC) and use-value (UV), were used to analyse the data.

*Use value (UV)* denotes the relative significance of species recognised locally^44^. The UV was used to identify the plants with the highest utilisation translating to the most frequently mentioned in the management of crop pests and diseases^45^. It was calculated as follows.

$$\:UV=\frac{Ui}{N}$$

where *Ui* is the number of uses stated by each participant for a specific species and *N* denotes the total number of participants. If a plant secures a high *UV* score, this indicates that there are many use reports for that plant, whereas a low score indicates fewer use reports cited by the participants.

*Relative frequency of citation (RFC)* as described by Tardío and Pardo-de-Santayana^42^, indicates the popularity of each species in the study area. This is obtained by dividing the number of participants who mention the plant, also known as frequency of citation (FC), by the number of participants included in the study (N). The RFC index was calculated as shown below.

$$\:RFC=\frac{\text{F}\text{C}}{\text{N}}$$

Where FC is the number of participants who reported using a certain species and N denotes the total number of participants in the research. The RFC has a value range of 0 to 1, with a high value indicating a high rate of popularity among the participants.

## Results and discussion

### Demographic characteristics of participants

The distribution of study participants by selected demographic characteristics including age, gender, marital status, employment status, and language is presented in Table 1. In this regard, the distribution of the participants by gender was assessed. Gender plays an important role in indigenous farming and crop management^46^. In this study, the distribution of the participants was approximately 35% and 65% for males and females, respectively (Table 1). The age of the study participants often influences the knowledge, attitude and practice of indigenous methods in crop management. The majority (27%) of the participants were aged 41–50 years old, while those within the other age groups accounted for ≤ 20% each of the study participants. The low rate of participation by those within the age group 18–30 years (14% in the current study) has been observed in other studies, and this may imply that the young generation engages less in farming activities^47,48^. Less interest shown by the youth may lead to the loss of indigenous knowledge. The unwillingness of the youth in engaging in these activities may mean that the knowledge sharing or passage to the new generations may be discontinued on the long run, thus leading to the loss of indigenous knowledge. Employment status is an important indicator of population capacity to earn income and provide for personal and family needs. In this regard, the employment status of participants is expected to influence their knowledge, attitudes and practices regarding indigenous farming and crop management. As indicated in Table 1, the majority (41%) of the study participants were unemployed, implying that they had no source of personal incomes.

**Table 1:** Socio-demographic characteristics of the participants in the selected communities for the study (*n* = 49).

Characteristic of participants	Category	Frequency	Percentage (%)
Age group (years)	18–30	7	14.3
	31–40	10	20.4
	41–50	13	27.5
	51–60	10	20.4
	61 and above	9	18.4
Gender	Male	17	35
	Female	32	65
Marital status	Married	16	33
	Single	31	63
	Divorced	2	4
Employment status	Student	1	2
	Employed, full-time	3	6
	Unemployed	20	41
	Self-employed	1	2
	Government social grant	5	10
	Other (please specify)	19	39
Language	Setswana	32	65.3
	Sesotho	7	14.3
	Other	10	20.4

### **Indigenous soil preparation and cropping systems**

Farmers in various regions of South Africa use diverse traditional methods including ethnopedology strategies, and the application of botanicals to protect crops against pests and diseases in the field and during storage^49,50^. These strategies often involve understanding soil health, crop rotation, and the ecological relationships within farming systems. The different methods and associated indigenous knowledge recorded in the current study are highlighted below.

#### Soil types in the three selected communities in Madibeng local municipality

The participants recruited from Madibeng local municipality understand that there are differences in soil-types in the study area. Four soil-types were reported in the communities of Madibeng local municipality (Table 2). An indigenous name of only one was mentioned, while the other three were described by their texture (Table 2). The soils found in the local municipality are sandy soils, loamy soil, silt soil, and clay soil (Table 2). In a study conducted by Kuria, Barrios, Pagella, Muthuri, Mukuralinda and Sinclair^51^, a similar trait was reported as participants described the soil types by looking at their texture and colour.

**Table 2:** Type of soils identified by the recruited participants in Madibeng local municipality of South Africa.

Soil-type	Local soil name/description	*N*	Percentage (%)
Sandy	Mmu o boleta	29	59.18
Loamy	Mmu o montsho	3	6.12
Slit	Mmu o matlapa	7	14.29
Clay	Seloko	10	20.41

N-number of mentions.

The participants explained that soil dynamics are complicated and therefore, they could not provide data on the testing of the soil acidity to determine whether the soil will be able to sustain crops. This was often tested through trial and error, with crop failure in a specific piece of land indicating that the soil was not good for cultivating plants. However, their great grandparents had the wisdom of determining whether the soil is too acidic to grow a plant or not by looking at the colour and feeling the texture with the fingers. The assessment may also entail tasting the soil to check its acidity level, which suggests the fertility. This aligns with the findings by Kuria, Barrios, Pagella, Muthuri, Mukuralinda and Sinclair^51^ who observed that farmers in two districts of Rwanda namely Nyabihu and Rabavu categorise the soil as of high or low quality using visual observations and touch which involves soil passing through fingers to assess soil texture, moisture content and ease of ploughing.

According to Gavrilescu^52^, naturally occurring water in soil is a crucial factor in promoting crop growth. Furthermore, Kirkham^53^ emphasised that water is the most important factor influencing plant growth, asserting that it is the primary environmental constraint limiting plant development. The participants in this study explained that, in addition to the gravitational water naturally present in the soil, they strategically water the plants to mitigate the effects of high temperatures and frequent heatwaves associated with the ongoing phase of climate change.

#### Soil preparation

Soil preparation is one of the most crucial stages in crop production^54,55^. The farmers in Madibeng local municipality use tools such as spades to turn and loosen the soil and to remove shrubs and weeds. This process is commonly the first stage of crop production in other developing countries including Nigeria, to cultivate staple crops (e.g., yam) and small tools including cutlasses are used for minimal soil disturbance^56,57^.

Digging is a crucial stage in crop production, and the length and width of the hole should be able to allow for proper root growth. The participants in this study explained that they dig using a shovel, while carefully making sure they measure the hole correctly. We identified a special tool that is used by 31% of the participants, which they made using metal piece (Supplementary Figure S1). The tool, assembled and welded by a well-known local community welder, is used to dig the ground for planting the seeds, and built in a way that it digs the appropriate depth that will allow the seeds or the roots (when transplanting) to penetrate the desired deep. The tool is used to measure the width and the depth of the hole where the seed will be placed.

The next step the participants of this study use in preparing the soil is to add manure into the soil and keep loosening it while watering it. This is done to ensure the manure penetrate deep into the soil so that the soil is well fertilised. According to Hettiarachchi^58^, clearing of shrubs before cultivating and allowing them to decompose after drying or if set on fire, allowing the ash to collect on the ground is an important practice in crop production. This is mainly because it loosens the soil while the burning process destroys weeds. Moreover, it increases soil fertility as well as the amount of humus in the topsoil, and it promotes the activities of soil organisms.

The waiting periods before planting the seeds varied among participants, with the duration ranging from 2 to 14 days. While 24% of the participants indicated reliance on rainfall, others were influenced by various other factors especially the lack of capital to buy seedlings in deciding when to plant. This variation in waiting periods aligns with findings from a previous study, which suggest that indigenous farmers determine the appropriate time for land preparation based on local rainfall patterns and the lunar calendar^59^. Additionally, 14% of the study participants noted that soil preparation duration in winter was longer compared to summer. During the waiting period, farmers regularly watered the plots, with some increasing the frequency to twice or even three times a day especially in the morning and afternoon when temperatures were particularly high.

Participants (8%) who waited for only two days after soil preparation explained that when planting occurs in the same area over time, the soil naturally loosens, reducing the need for an extended waiting period. In contrast, those who waited up to two weeks (67%) emphasised the importance of allowing the soil to loosen sufficiently to enable the roots to grow deeply. They believed that this was a crucial stage that helps with preventing crop failure. Participants who waited for rain highlighted the need for adequate water for the crops, stressing that waiting for rain was essential to ensure proper growth. The participants emphasised the importance of rain for their farms as they do not have access to other water sources.

Soil preparation methods would vary across different seasons, but participants in this study explained that they consistently use the same preparation techniques each time they plant. They mentioned two primary reasons for this: first, these methods are the only ones they know, having been passed down from their parents and grandparents. In addition, these methods have proven effective across seasons, consistently yielding high crop production. According to the participants: *“Fa o jala dijo tse di tlhogang mariga ka nako ya mariga di tlile go tswa sentle go se kgathalesege gore o baakanyeditse jang mmu wa gago (translated as “If you plant winter sprouts in the winter*,* they will do well no matter how you prepare your soil)”.* The statement was made by participants to emphasise the importance of growing appropriate crops in their season to prevent crop failure.

#### Indigenous practices used to enhance soil fertility

In this study, livestock (goat, pig, cow, and poultry) waste/manure and plant residues (e.g., dry grasses) were often applied to enhance soil fertility. The participants alluded to several reasons for the popularity of these products in the study area. These products are readily available within the village, which means they are cost-effective, and they are good natural fertilisers of the soil. In addition, these waste materials are considered safe for the soil when they are used correctly. Most participants (92%) explained that the application of manure enhances the resultant yields of fresh and healthy vegetables. Although similar good yield is associated with the application of chemical fertilisers, the undesired damage to the soil and residual effect over time remain a concern. This disposition aligns with the reasons given by participants in other studies focusing on farmers in indigenous communities^60–62^.

The participants emphasised that the animal waste must be in their dry state when mixed into the soil. This is mainly because mixing with the soil while it is still wet will burn the root of the crops and kill them. The drying process in indigenous community uses sun-drying while in commercial agriculture, the process is done through the use of technological tools such as industrial drying facility^63^. According to Bhunia, Bhowmik, Mallick and Mukherjee^64^, drying livestock waste has the potential to eliminate infectious pathogens from the final product.

The grasses must be dried so that it can easily decompose and mix with the soil. The indigenous farmers in rural communities are often characterised by marginalisation and limited capital^65^. In this study, more than 80% of the participants identified this natural manure as readily available within their environment. While some farmers with livestock are more privileged, others ask for the manure from their neighbours who own livestock. This is a practice that has been long standing in the study area, showing that the philosophy of Ubuntu (humanity) still plays a role in the livelihoods of people in rural communities.

In the study area, plant materials such as leaves and twigs of trees or shrubs, and cultivated legume crops are used for producing the green manure by the local farmers. These can be turned during the tillage process or ploughed into the soil to enhance soil structure and soil fertility. Besides the fact that green manure adds organic matter and nutrients to the soil, which helps in the maintenance of soil fertility needed for maximum crop growth and higher yields, the humus formed from it enhances water holding capacity of the soil, decreases soil loss by erosion, conserves moisture and prevents nutrient leeching^66^.

Aligned with the application of green manure, there is increasing evidence that degraded land can be restored and kept productive. This can be achieved through a process called perennialization where perennial crops and forages are incorporated in long rotations^67,68^. The local farmers in Madibeng local municipality also use ash from certain tree branches to fertilise their soils for crop production. In most other areas, this is done through the slash and burn process where the field is burnt, and the resulting ash is used to fertilise the soil. However, this practice is slowly being discarded as it is perceived to have some negative implications on the environment^69–71^. Likewise, Mebrate, Zeray, Kippie and Haile^72^ reported that farmers in Gedeo zone in Southern Ethiopia depend on organic soil fertility practices instead of chemical fertilisers as a source of nutrients. In the study by Mebrate, Zeray, Kippie and Haile^72^, the majority of the farmers use green manure to increase soil health.

According to Rowen, Tooker and Blubaugh^73^, managing soil fertility is the key to maintaining soil quality in crop systems and can have significant implications on crop growth and insect pest infestation. The authors explained that the use of organic fertilisers is hypothesised to promote plant growth, herbivore resistance and pest suppression. This view was corroborated by Urriago-Ospina, Jardim, Rivera-Fernandez, Kozovits, Leite and Messias^74^, who reported that organic manure is widely used and can be categorised into two main types: organic residues, and manure addition. These categories encompass household waste and livestock-derived products.

Sustainability in agriculture and the maintenance of soil quality are highly dependent on healthy productive soils and this often involves active soil management with carefully chosen soil fertility amendments^75,76^. The authors asserted that animal waste can deliver a variety of nutrients and enhance microbial activity that can increase crop productivity. The use of livestock waste to increase soil health is practiced in other southern Africa countries such as Botswana, Zimbabwe and Zambia^77–79^. In a study conducted by Abeywardana, Schütt, Wagalawatta and Bebermeier^80^, the local farmers in the dry zone of Sri Lanka allow cattle to graze in the fields they use for crop production after the crops have been harvested, and by so doing, the secreted cow dung and urine that fall onto the field are used as a natural fertiliser.

According to Verma and Verma^81^, livestock waste from animals such as cattle, sheep, goats and chickens are good source of organic manure with high nutrients. Chicken manure is particularly reported by the authors as extremely rich in nitrogen and organic matter, and suitable for all crops. The application of these materials can also be a solution to top soil erosion^82^. In Gedeo located in Ethiopia, soil acidity was identified as the greatest challenge. To amend soil fertility challenges, farmers adopted the application of organic manure mainly from cows and horses to their soils^83^.

### Cropping systems used by local farmers

The local farmers in Madibeng local municipality mainly use two cropping systems namely mixed cropping/intercropping and crop rotation. These cropping systems are also used in other parts of the world including India^84^. The local farmers explained that they use these cropping systems mainly because of their benefits to the soil and to them as the growers and the consumers of the crops. The cropping systems offer dietary diversity in a sense that a variety of food crops is available at the same time to meet their nutritional needs. These cropping systems also decreased the spread of diseases as a different crop is planted next to another crop, thus limiting the spread of diseases.

According to Rankoana^85^, intercropping/mixed cropping can help to maintain soil conditions and fertility, control weeds and pests. With this cropping system, the productivity tends to be higher than mono-cropping. The participants in this study explained that mixed cropping also reduces the chances of crop failure. This is supported by Nasar, Alam, Nasar and Khan^86^ who indicated that mixed cropping enhances crop production and returns as well as prevent complete crop failure. The authors explained that intercropping system maximizes the available plant growth resources such as water, sunlight, and nutrients, and can minimise competition with weeds, and attacks by pests and diseases.

The intercropping process is not done in any specific order, and the participants did not have any criteria on selecting crops to be intercropped. Generally, wild garlic (*Tulbaghia violaceae* Harv.) is often intercropped for pesticidal purpose by the farmers in the study area. In other local communities, farmers intercrop with crops that complement each other to eliminate competition for resources especially soil nutrients^87^. The local farmers in Madibeng local municipality use the trial-and-error method and observe the crop growth performance to identify crops suitable for their specific soil types. The crop rotation helps with maintaining the soil quality and allows for crop productivity annually without failure^88–90^. Similarly, crop rotation and intercropping are used among the Vhavenda in Musina local municipality, South Africa as sustainable indigenous methods of crop production^91^.

In the study area, various crops were cultivated by farmers which contribute to the diversification of their diet and food security. The commonly cultivated crops in the backyards include spinach, carrots, tomatoes, onions, cabbage (red and green), beetroot, sweet potato, butternut, green pepper, lettuce, butternut and okra. These crops are not necessarily indigenous crops, but they are consumed in this area due to preference. However, in other indigenous communities, local farmers cultivate staple crops that are indigenous to their respective communities^92,93^.

These crops are consumed by the local farmers and members of their households. In some cases, the extra produce is sold in local markets. The participants who are involved with the sale of the cultivated crops indicated that they chose to grow these crops because of the demand in the communities. The participants also demonstrated an understanding of the essential nutrients that most of these food crops have and the role they play in keeping them healthy. Most of the crops that are planted are vegetables, which are recommended for healthy diets^94–96^. In this study, the participants believed that they grow these crops organically, without the fear of residue effect of chemicals that may negatively affect their health when consumed. Generally, organic foods are considered or perceived as safer and healthier than genetically modified food or food cultivated using agro-chemicals^93,97^.

### Indigenous crop pest and disease management strategies

Traditional pest and disease management have been developed over generations and used by local farmers for centuries^15^. In Africa, diverse traditional methods, including ethnopedology strategies are explored for protecting crops against pests and diseases both in the field and during storage^50,98^. These strategies often involve understanding soil health, crop rotation, and the ecological relationships within farming systems. Some of the major methods and associated indigenous knowledge, which were evident in the study area are presented below.

#### Utilisation of botanicals for the management of crop pests and diseases

We identified 10 plant species belonging to 7 families that were used in the management of 5 pests and 7 diseases affecting different crops (Table 3). The RFC indicates the local importance of plant species with reference to the participants, who cited the uses of these plants^42^. In the study, the RFC ranged from 0.06 to 0.88 for the recorded 10 plants. Based on the RFC, the most cited plant species were *Allium dregeanum* Kunth (0.88), *Tulbaghia violacea* Harv. (0.73), *Capsicum frutescens* L. (0.63) and *Vachellia tortilis* (Forssk.) Galasso & Banfi (0.59) (Table 3). Most of these plants are regarded as popular botanicals and forms part of the local inventory as an environmentally friendly, accessible, and affordable alternative for crop protection. For instance, *Tulbaghia violacea* was among the most cited plant species for crop protection in studies conducted across different provinces including the Eastern Cape and Mpumalanga^50,98^.

The use-value (UV) is a measure of the type of uses attributed to a particular plant species. In the present study, *Tulbaghia violacea* Harv. had the highest UV (0.1), followed by *Aloe greatheadii* var. *davyana* (Schonland) Glen & D.S Hardy, *Capsicum frutescens* L., *Combretum imberbe* Wawra, *Peltophorum africana* Sond. and *Vachellia tortilis* (forssk) Galasso & Banfi UV (0.06) (Table 3). On the other hand, it is important to note that plants with lower UV and RFC values are not necessarily less important. Rather, the low ethnobotanical indices may reflect a lack of awareness among the participants regarding the potential uses of the plants. This lack of recognition presents a risk to the transmission of knowledge about these plants, which may not be passed down to future generations. As a result, the understanding of these plants and their applications could diminish over time, leading to the loss of valuable traditional knowledge. To safeguard this knowledge and explore their potential, these plants should undergo pharmacological, phytochemical, and biological investigations to assess their therapeutic properties and the possibility of developing low-cost products^99^.

The importance of trees/shrubs among local communities has been documented in countries such as South Africa, Namibia, Zimbabwe Ethiopia, India, Estonia and Indonesia^100–107^. These studies suggest that local communities often rely on locally sourced plants for diverse uses and as a source of livelihood. The life forms that were cited the most by the local farmers in Madibeng local municipality are trees/shrubs. All the plants were documented with their local names, most of which correspond to Setswana names. In terms of the life form of the documented plants, they included 6 trees/shrub, 3 herb, and 1 succulent. Among the plants, 70% were indigenous/native while 30% were naturalised (Table 3). Fabaceae with 3 plant species and Alliaceae with two plant species were the dominant plant families.

Crops are affected by various diseases and pests that hinder their growth or even cause crop failure. Crop diseases and pests reduce agricultural production and increase food insecurity from household to global levels^108^. In the study area, the issue of crop pests and diseases remains a major problem. Although they do not have specific names for these diseases and pests, they often describe them by their colour, shape, or pest category, for example, *‘’seboko se se tala’’* which directly translates to green worm. Similar approach was reported in two rural districts of Tanzania in a study conducted by Laizer, Chacha and Ndakidemi^109^, whereby the participants were able to identify pests through describing them using pictorial aid.

In Madibeng local municipality, the participants who are indigenous farmers reported that they have relied primarily on the use of plants to protect their crops from pests and diseases for many years. This practice aligns with the findings of Abate, van Huis and Ampofo^23^, who indicated that traditional pest control methods remain the most widely used approaches in Africa. The authors further explained that, although farmers have used plants for crop management for generations, the active compounds in these plants were not always known, and the application methods were culturally dependent. Additionally, these botanical solutions are both accessible and cost-effective.

**Table 3:** Overview of plants used for protection against crop pests and diseases among local farmers in Madibeng local municipality, South Africa.

*Scientific name (Voucher number)	Plant family	Local name	FC	UV	RFC	Diseases	Pests	Plant part used, preparation mode and administration methods and (dosage level)	Target crop(s)	Life form
*Allium dregeanum Kunth (KT5)	Alliaceae	Eie	43	0.04	0.88	Bacterial spot, powdery, mildew, club root	Worms, spiders	Bulb is cut into small pieces and boiled in water and then sprayed on top of the crop four times a week (1 L)	Spinach, cabbage, corn, potatoes, tomatoes, carrot, beetroot, beans, cabbage, green pepper, lettuce, cucumber	Herb
*Aloe greatheadii var.davyana (schonland) glen & D.S. Hardy (KT 6)	Asphodelaceae	Kgopane ya fatshe	13	0.06	0.27	Early blight	Worms, ants	Whole plant cut into small pieces, mixed with sunlight bar soap, boiled in water, sprayed around crop four times a week (1 L)	Spinach, cabbage, corn, potatoes, carrot, beetroot, beans, green pepper	Succulent
*Cannabis sativa* L. var. sativa (KT 7)	Cannabaceae	Matekwane	7	0.04	0.14	Bacterial spot	Not given	Plant seed in between crops (Intercropping) (one plant species between two crops)	Spinach, cabbage	Shrub
*Capsicum frutescens* L. (KT 9)	Solanaceae	Pherefere	31	0.06	0.63	Powdery mildew, clubroot	Locust	Cut plant into small pieces, boil in water and the spray around the crop four times a week (1 L)	Cabbage, spinach, beetroot, tomatoes, lettuce	Herb
*Combretum imberbe Wawra (KT 8)	Combretaceae	Mohwilire	3	0.06	0.06	Not given	Worms	Burn the bark and surf out the ash then sprinkle around the crop, twice (handful of ash around crop) a month	Spinach, corn, tomatoes, carrot, beetroot, beans, cabbage, green pepper	Tree
*Dichrostachys cinerea* (L.) wigt & arn. (KT 3)	Fabaceae	Moselesele	12	0.08	0.24	Powdery mildew, clubroot	Locust	Burn the bark and surf out the ash then sprinkle around the crop, twice a month (handful of ash around crop)	Spinach, cabbage, beetroot, tomatoes, cucumber, lettuce	Tree
*Peltophorum africana Sond. (KT 4)	Fabaceae	Mosetlha	4	0.06	0.08	Early leaf spot of groundnut, downy mildew, maize ear rot	Locust	Burn the bark and surf out the ash then sprinkle around the crop, twice a month (handful of ash around crop)	Groundnut, cabbage, maize	Tree
*Tulbaghia violaceae harv (KT 10)	Alliaceae	Konofole	36	0.1	0.73	Bacterial spot, powdery mildew	Worms, spiders, termites	Whole plant is cut into small pieces and boiled in water and then sprayed on top of the crop four times a week (1 L)	Spinach, cabbage, corn, potatoes, tomatoes, carrot, beetroot, beans, green pepper, pumpkin	Herb
*Vachellia tortilis (forssk.) Galasso & Banfi (KT 2)	Fabaceae	Mosunyane	29	0.06	0.59	Powdery mildew	Locust	Burn the bark and surf out the ash then sprinkle around the crop, twice a month (handful of ash around crop)	Cabbage	Tree
*Ziziphus mucronata Willd. Subsp. Mucronata (KT 1)	Rhamnaceae	Mokgalo	10	0.06	0.2	Bacterial spot, early leaf of groundnut	Locust	Burn the bark and surf out the ash then sprinkle around the crop, twice a month (handful of ash around crop)	Spinach, groundnut, beetroot, tomatoes, lettuce	Tree

FC = frequency of citation, uv = use value, rfc = relative frequency of citation. *Plants with asterisk are considered indigenous or naturalised to South Africa.

According to Dar, Bhat, Mehmood and Hakeem^110^, the use of pesticides is being reduced in agricultural production because they also harm the soil organisms and may leave toxic residues in crops. The authors also alluded to the indirect harm pesticides cause to aquatic life. As reported by the participants, the use of certain trees to manage crop pests and diseases is an old practice. The trees were preferred because their ash was not harmful to crops.

**Fig. 2:**
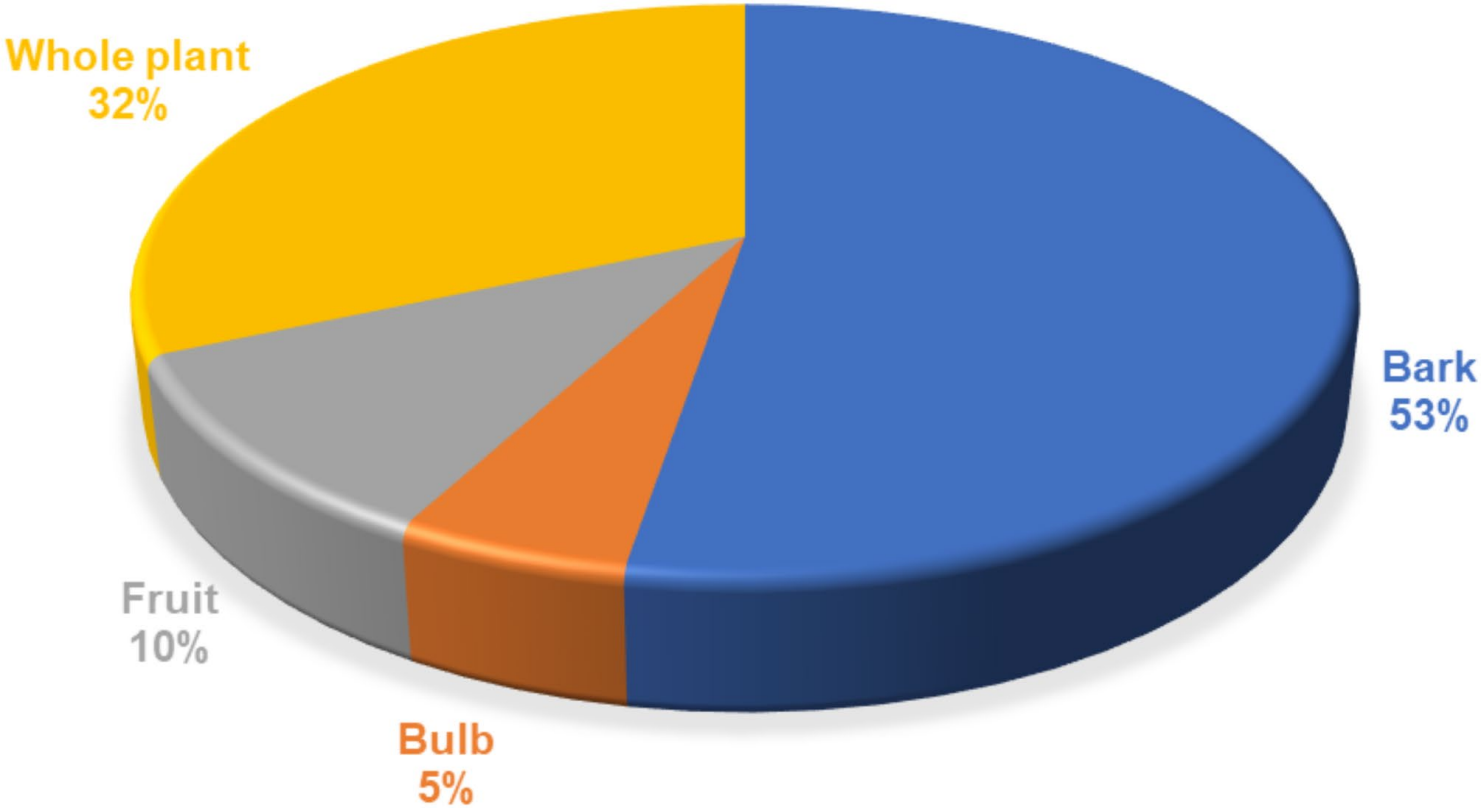
A representation of plant parts used in preparation of remedies for the protection of crop pests and diseases.

In the study area, different plant parts were used for the preparation of remedies for the protection of crop pests and diseases (Fig. 2). The most dominant plant parts used were bark (53%) and whole plant (32%). The use of different plant parts to manage crop pests and diseases is common in other studies in South Africa^49,98^ and Nigeria^111^. However, in most studies, the leaves were generally the most used plant part^49,98^.

In this study, farmers worked with a variety of plant parts in the preparation of remedies for the management of crop pests and diseases. To prepare plant materials, farmers used different formulations and combinations prepared in the form of ashing (50%), decoction (30%) and direct intercropping (10%) (Table 3). Farmers in Madibeng also use administration methods such as ash scattering (50%), spraying (40%) and direct intercropping (10%). The use of formulations and combinations by farmers in these study areas for insect pest management is consistent with small-scale farmers from other parts of South Africa and Africa^112,113^. Water is mostly used to dilute the juice from the plant. Few remedies are prepared from dried and ground plant parts. Most of the remedies were reported as being prepared from a single plant species.

## Conclusion

This study revealed that local farmers in Madibeng local municipality use indigenous farming methods and practices for crop production and food security. The participants identified four types of soil in Madibeng local municipality. Participants indicated that they plant crops in all these soil types. In this study, the duration of soil preparation before planting varies with the local farmers in Madibeng local municipality. The study also identified five soil fertilising materials from two main sources namely, livestock (cow dung, poultry manure, pig waste, goat waste) and plants (dry grass) that are utilised by the local farmers. The prominent cropping systems in Madibeng local municipality are mixed cropping/intercropping and crop rotation. These cropping systems have allowed them to continue to cultivate several plants in their yards on the same plot/space for many years. In this study, 10 plant species were identified as natural resources used for crop protection in Madibeng local municipality. The continuous use of botanicals to manage crop pests and diseases shows their potential in sustainable crop pest and disease management. Indigenous farmers have significant knowledge on sustainable use of natural resources. Taken together, it is evident that the applications of indigenous farming practices and methods is pertinent for food security among rural communities. Efficient approaches aimed at incorporating community-based valuable indigenous knowledge in modern agricultural practice is recommended as a means of meeting the targets associated with zero hunger especially among local communities.

## Electronic supplementary material

Below is the link to the electronic supplementary material.

Supplementary Material 1.

## Data Availability

Data used for this study have been included as part of the article.

## References

[1] Patel, S. K., Sharma, A. & Singh, G. S. Traditional agricultural practices in India: An approach for environmental sustainability and food security. *Energy Ecol. Environ.* **5**, 253–271. 10.1007/s40974-020-00158-2 (2020).

[2] Rankoana, S. A. Indigenous knowledge and innovative practices to cope with impacts of climate change on small-scale farming in Limpopo Province, South Africa. *Int. J. Clim. Change Strateg. Manag.* **14**, 180–190 (2022).

[3] Ogen, O. Traditional farming and Indigenous knowledge systems in Africa: Perspective from the Ikale-Yoruba experience. *Indilinga Afr. J. Indigenous Knowl. Syst.* **5**, 157–166 (2006).

[4] Vilakazi, B. S., Zengeni, R. & Mafongoya, P. Indigenous strategies used by selected farming communities in KwaZulu-Natal, South Africa, to manage soil, water, and climate extremes and to make weather predictions. *Land. Degrad. Dev.* **30**, 1999–2008. 10.1002/ldr.3395 (2019).

[5] Rankoana, S. A. The use of indigenous knowledge in subsistence farming: implications for sustainable agricultural production in Dikgale community in Limpopo Province, South Africa. in *Towards Sustainable Agriculture Farming Practices Water Use*. *Frontiers in Sustainability*, Vol. 1. 63. 10.3390/books978-3-03842-331-7-4 (2017).

[6] Chanza, N. & Musakwa, W. Revitalizing Indigenous ways of maintaining food security in a changing climate: Review of the evidence base from Africa. *Int. J. Clim. Change Strateg. Manag.* **14**, 252–271. 10.1108/IJCCSM-06-2021-0065 (2022).

[7] De Bruin, F. M. Exploring the benefits and challenges of indigenous foods in an African context using a case study of community gardens in the Western Cape of South Africa. (Master of Philosophy in Sustainable Development in the Faculty of Economic and Management Sciences at Stellenbosch University, South Africa, 2018).

[8] Baiphethi, M. N. & Jacobs, P. T. The contribution of subsistence farming to food security in South Africa. *Agrekon* **48**, 459–482 (2009).

[9] Borelli, T. et al. Born to eat wild: An integrated conservation approach to secure wild food plants for food security and nutrition. *Plants* **9**, 1299. 10.3390/plants9101299 (2020). 33019632 10.3390/plants9101299 PMC7601573

[10] Hoffman, M. T. & Todd, S. A National review of land degradation in South Africa: The influence of biophysical and socio-economic factors. *J. South. Afr. Stud.* **26**, 743–758. 10.1080/713683611 (2000).

[11] van Huyssteen, C. W., du Preez, C. C. & Holmes, P. J. Agriculture and a changing biophysical environment. in *Southern African Landscapes and Environmental Change* (eds Holmes, P. J. & Boardman, J.) 228–248 (Routledge, 2018).

[12] Maila, M. W. & Loubser, C. P. Emancipatory Indigenous knowledge systems: Implications for environmental education in South Africa. *South. Afr. J. Educ.* **23**, 276–280 (2003).

[13] Jessen, T. D., Ban, N. C., Claxton, N. X. & Darimont, C. T. Contributions of indigenous knowledge to ecological and evolutionary understanding. *Front. Ecol. Environ.* **20**, 93–101. 10.1002/fee.2435 (2022).

[14] Egeruoh-Adindu, I. Leveraging Indigenous knowledge for effective environmental governance in West Africa. *Beijing Law Rev.* **13**, 931. 10.4236/blr.2022.134060 (2022).

[15] Melash, A. A. et al. Indigenous agricultural knowledge: A neglected human based resource for sustainable crop protection and production. *Heliyon* **9**(23), e12978. 10.1016/j.heliyon.2023.e12978 (2023). 36711305 10.1016/j.heliyon.2023.e12978 PMC9876958

[16] Hlophe-Ginindza, S. N. & Mpandeli, N. S. The role of small-scale farmers in ensuring food security in Africa. in *Food Security in Africa* (ed Mahmoud, B.), Vol. 1. 10.5772/intechopen.91694 (2021).

[17] Solomon, D. et al. Indigenous African soil enrichment as a climate-smart sustainable agriculture alternative. *Front. Ecol. Environ.* **14**, 71–76. 10.1002/fee.1226 (2016).

[18] Reijntjes, C., Haverkort, B. & Waters-Bayer, A. *Farming for the Future. An Introduction To low-external-input and Sustainable Agriculture* (Macmillan Educ., 1992).

[19] Misra, S. & Ghosh, A. Agriculture paradigm shift: a journey from traditional to modern agriculture. in *Biodiversity and Bioeconomy: Status Quo, Challenges, and Opportunities* (eds Singh, K. et al.) 113–141 (Elsevier, 2024).

[20] Nhamo, L. et al. Cereal production trends under climate change: Impacts and adaptation strategies in Southern Africa. *Agriculture* **9**, 30. 10.3390/agriculture9020030 (2019).

[21] Giller, K. E. et al. The future of farming: Who will produce our food? *Food Security* 13, 1073–1099. 10.1007/s12571-021-01184-6 (2021).

[22] Muimba-Kankolongo, A. *Food Crop Production by Smallholder Farmers in Southern Africa: Challenges and Opportunities for Improvement* (Andre Gerhard Wolff, 2018).

[23] Abate, T., van Huis, A. & Ampofo, J. Pest management strategies in traditional agriculture: An African perspective. *Ann. Rev. Entomol.* **45**, 631–659. 10.1146/annurev.entro.45.1.631 (2000). 10761592 10.1146/annurev.ento.45.1.631

[24] Mugambiwa, S. S. Adaptation measures to sustain Indigenous practices and the use of Indigenous knowledge systems to adapt to climate change in Mutoko rural district of Zimbabwe. *Jàmbá: J. Disaster Risk Stud.* **10**, a388. 10.4102/jamba.v10i1.388 (2018). 10.4102/jamba.v10i1.388 PMC6014056 29955251

[25] Kom, Z., Nicolau, M. D. & Nenwiini, S. C. The use of indigenous knowledge systems practices to enhance food security in Vhembe District, South Africa. *Agric. Res.* 13, 599–612. 10.1007/s40003-024-00716-8 (2024).

[26] Shilomboleni, H., Epstein, G. & Mansingh, A. Building resilience in Africa’s smallholder farming systems: Contributions from agricultural development interventions—a scoping review. *Ecol. Soc.* **29**, 22. 10.5751/ES-15373-290322 (2024).

[27] Altieri, M. A. Linking ecologists and traditional farmers in the search for sustainable agriculture. *Front. Ecol. Environ.* **2**, 35–42. 10.1890/1540-9295(2004)002[0035:LEATFI]2.0.CO;2 (2004).

[28] Muhie, S. H. Novel approaches and practices to sustainable agriculture. *J. Agric. Food Res.* **10**, 100446. 10.1016/j.jafr.2022.100446 (2022).

[29] Khatri, P., Kumar, P., Shakya, K. S., Kirlas, M. C. & Tiwari, K. K. Understanding the intertwined nature of rising multiple risks in modern agriculture and food system. *Environ. Dev. Sustain.* 26, 24107–24150. 10.1007/s10668-023-03638-7 (2024).

[30] Ba, Q. X., Lu, D. J., Kuo, W. H. J. & Lai, P. H. Traditional farming and sustainable development of an Indigenous community in the mountain area—a case study of Wutai village in Taiwan. *Sustainability* **10**, 3370. 10.3390/su10103370 (2018).

[31] Pilane, M. V. Public Participation in the Integrated Development Plan: A Case of Madibeng Local Municipality, North-West. (Master of Management degree in Public Policy at the University of Witwatersrand, Johannesburg, South Africa, 2023).

[32] John, J., Pieterse, A., Chilwane, L. & van Niekerk, W. Madibeng Local Municipality: Adaptation Action Plan, (2024).

[33] Google Map, n.d. Madibeng local communities. Accessed 31 Mar 2024. Available from https://maps.app.goo.gl/cTxadJy7dVwBhLS8A.

[34] Tefera, B., Ruelle, M. L., Asfaw, Z. & Abraha Tsegay, B. Woody plant diversity in an Afromontane agricultural landscape (Debark district, Northern Ethiopia). *For. Trees Livelihoods* **23**, 261–279. 10.1080/14728028.2014.942709 (2014).

[35] Creswell, J. W. *Research methods*. Research design: Qualitative, quantitative and mixed methods approaches (eds Laughton, C. N. & Creswell, J. W.) 15–17 (Sage Publications, London, New Delhi, 2009).

[36] Adams, R. S., Daly, S. R., Mann, L. M. & Dall’Alba, G. Being a professional: Three lenses into design thinking, acting, and being. *Des. Stud.* **32**, 588–607. 10.1016/j.destud.2011.07.004 (2011).

[37] Alexiades, M. & Sheldon, J. *Selected Guidelines for Ethnobotanical Research: A Field Manual, 1996* (The New York Botanical Garden, New York Botanical Garden New York, USA, 1996).

[38] Heckathorn, D. D. Respondent-driven sampling: a new approach to the study of hidden populations. *Soc. Probl.* **44**, 174–199. 10.2307/3096941 (1997).

[39] Kish, L. On the future of survey sampling. *Surv. Sampl. Meas.* 13–21 (1978).

[40] Heckathorn, D. D. Comment: snowball versus respondent-driven sampling. *Sociol. Methodol.* **41**, 355–366. 10.1111/j.1467-9531.2011.01244.x (2011). 22228916 10.1111/j.1467-9531.2011.01244.x PMC3250988

[41] Bless, C., Higson-Smith, C. & Kagee, A. in *Fundamentals of Social Research Methods: An African Perspective* (ed. Bless, C. A.) 1–186 (Juta and Company Ltd, Lusaka, 2006).

[42] Tardío, J. & Pardo-de-Santayana, M. Cultural importance indices: a comparative analysis based on the useful wild plants of Southern Cantabria (Northern Spain). *Econ. Bot.* **62**, 24–39. 10.1007/s12231-007-9004-5 (2008).

[43] Phillips, O. & Gentry, A. H. The useful plants of Tambopata, Peru: I. Statistical hypotheses tests with a new quantitative technique. *Econ. Bot.* **47**, 15–32 (1993). https://www.jstor.org/stable/4255479

[44] Vitalini, S. et al. Traditional knowledge on medicinal and food plants used in Val San Giacomo (Sondrio, Italy)—An alpine ethnobotanical study. *J. Ethnopharmacol.* **145**, 517–529. 10.1016/j.jep.2012.11.024 (2013). 23220197 10.1016/j.jep.2012.11.024

[45] Hudaib, M. et al. Ethnopharmacological survey of medicinal plants in Jordan, mujib nature reserve and surrounding area. *J. Ethnopharmacol.* **120**, 63–71. 10.1016/j.jep.2008.07.031 (2008). 18760342 10.1016/j.jep.2008.07.031

[46] Gwandure, C. & Lukhele-Olorunju, P. Women’s use of indigenous knowledge in Africa. *Social Sci. Humanit. Open.* **8**, 10074. 10.1016/j.ssaho.2023.100741 (2023).

[47] Buthelezi, N., Hughes, J. & Modi, A. The use of scientific and Indigenous knowledge in agricultural land evaluation and soil fertility studies of two villages in KwaZulu-Natal, South Africa. *Afr. J. Agric. Res.* **8**, 507–518 (2013).

[48] Taye, A. & Megento, T. L. The role of Indigenous knowledge and practice on water and soil conservation management in Albuko woreda, Ethiopia. *Int. J. Bonorowo Wetl.* **7**, 95–107. 10.13057/bonorowo/w070206 (2017).

[49] Shai, K. N., Chakale, M. V., Materechera, S. A., Amoo, S. O. & Aremu, A. O. Utilisation of botanicals for the management of pests and diseases affecting crops in sub-Saharan Africa: A review. *J. Nat. Pesticide Res.* **7**, 100066. 10.1016/j.napere.2023.100066 (2023).

[50] Shai, K. N., Materechera, S. A., Amoo, S. O. & Aremu, A. O. Ethnobotanical insights on the management of plant pests and diseases by smallholder farmers in Mpumalanga Province of South Africa. *J. Ethnobiol. Ethnomed.* **20**, 71. 10.1186/s13002-024-00711-x (2024). 39085935 10.1186/s13002-024-00711-x PMC11293110

[51] Kuria, A. W. et al. Farmers’ knowledge of soil quality indicators along a land degradation gradient in Rwanda. *Geoderma Reg.* **16**, e00199. 10.1016/j.geodrs.2018.e00199 (2019).

[52] Gavrilescu, M. Water, soil, and plants interactions in a threatened environment. *Water* **13**, 2746. 10.3390/w13192746 (2021).

[53] Kirkham, M. B. *Principles of Soil and Plant Water Relations* (Academic, 2023).

[54] Lal, R. Soils and sustainable agriculture: A review. in *Sustainable Agriculture* (eds Lichtfouse, E. et al.) (Springer Nature, 2009).

[55] Bot, A. & Benites, J. *The Importance of Soil Organic Matter: Key To drought-resistant Soil and Sustained Food Production* (Food & Agriculture Organisation, 2005).

[56] Ema, E. O. S., Obidiegwu, J. E., Chilaka, C. A. & Akpabio, E. M. Indigenous food yam cultivation and livelihood practices in Cross River State, Nigeria. *World* **4**, 314–332. 10.3390/world4020020 (2023).

[57] Sofiyuddin, M., Suyanto, S., Kadir, S. & Dewi, S. Sustainable land preparation for farmer-managed lowland agriculture in Indonesia. *For. Policy Econ.* **130**, 102534. 10.1016/j.forpol.2021.102534 (2021).

[58] Hettiarachchi, H. Application of indigenous knowledge in rural agriculture: A study on existing Indigenous practices among women farmers in Monaragala district, Sri Lanka. *Int. J. Multidisciplinary Res. Dev.* **9**, 46–56 (2022).

[59] Irangani, M. K. L. S. Indigenous techniques used in rice cultivation in Sri Lanka: An analysis from and agricultural history perspective. *Indian J. Tradit. Knowl.* **12**, 638–650 (2013).

[60] Carr, P. M. et al. Green and animal manure use in organic field crop systems. *Agron. J.* **112**, 648–674. 10.1002/agj2.20082 (2020).

[61] Brust, G. E. Management strategies for organic vegetable fertility. in *Safety and Practice for Organic Food* (eds Biswas, D. & Micallef, S. A.) 193–212. 10.1016/B978-0-12-812060-6.00009-X (Academic Press, Cambridge, 2019).

[62] Sajjad, M. R. et al. Performance of green manuring for soil health and crop yield improvement. *Pure Appl. Biol.* **8**, 1543–1553. 10.19045/bspab.2019.80095 (2019).

[63] Bhunia, S., Bhowmik, A. & Mukherjee, J. *International Conference on Energy Management for Green Environment* (Institute of Electrical and Electronics Engineers, Kolkata, India, 2019).

[64] Bhunia, S., Bhowmik, A., Mallick, R. & Mukherjee, J. Agronomic efficiency of animal-derived organic fertilizers and their effects on biology and fertility of soil: A review. *Agronomy* **11**, 823. 10.3390/agronomy11050823 (2021).

[65] Neves, D. & Du Toit, A. Rural livelihoods in South Africa: Complexity, vulnerability and differentiation. *J. Agrar. Change* **13**, 93–115. 10.1111/joac.12009 (2013).

[66] Singh, T. B. et al. Role of organic fertilizers in improving soil fertility. in *Contaminants in Agriculture: Sources, Impacts and Management* (eds Neem M. G. & Ansari, A. A.) 61–77 (Springer Nature Switzerland AG, Switzerland, 2020).

[67] Bell, S. M., Barriocanal, C., Terrer, C. & Rosell-Melé, A. Management opportunities for soil carbon sequestration following agricultural land abandonment. *Environ. Sci. Policy* **108**, 104–111. 10.1016/j.envsci.2020.03.018 (2020).

[68] Mosier, S., Córdova, S. C. & Robertson, G. P. Restoring soil fertility on degraded lands to Meet food, fuel, and climate security needs via perennialization. *Front. Sustainable Food Syst.* **5**, 706142. 10.3389/fsufs.2021.706142 (2021).

[69] Fajrini, R. Environmental harm and decriminalization of traditional slash-and-burn practices in Indonesia. *Int. J. Crime. Justice Soc. Democr.* **11**, 28–43. 10.3316/informit.379398893195786 (2022).

[70] Kukla, J. et al. The effect of traditional slash-and‐burn agriculture on soil organic matter, nutrient content, and microbiota in tropical ecosystems of Papua new Guinea. *Land. Degrad. Dev.* **30**, 166–177. 10.1002/ldr.3203 (2019).

[71] Hands, M. The search for a sustainable alternative to slash-and-burn agriculture in the world’s rain forests: The Guama model and its implementation. *Royal Soc. Open. Sci.* **8**, 201204. 10.1098/rsos.201204 (2021). 10.1098/rsos.201204 PMC8074668 33972850

[72] Mebrate, A., Zeray, N., Kippie, T. & Haile, G. Determinants of soil fertility management practices in Gedeo zone, Southern Ethiopia: Logistic regression approach. *Heliyon* **8**, e08820 (2022). 35128105 10.1016/j.heliyon.2022.e08820 PMC8803585

[73] Rowen, E., Tooker, J. F. & Blubaugh, C. K. Managing fertility with animal waste to promote arthropod pest suppression. *Biol. Control* **134**, 130–140. 10.1016/j.biocontrol.2019.04.012 (2019).

[74] Urriago-Ospina, L. M. et al. Traditional ecological knowledge in a ferruginous ecosystem management: lessons for diversifying land use. *Environ. Dev. Sustain.* **23**, 2092–2121. 10.1007/s10668-020-00665-6 (2021).

[75] Bonanomi, G., Lorito, M., Vinale, F. & Woo, S. L. Organic amendments, beneficial microbes, and soil microbiota: Toward a unified framework for disease suppression. *Annu. Rev. Phytopathol.* **56**, 1–20. 10.1146/annurev-phyto-080615-100046 (2018). 29768137 10.1146/annurev-phyto-080615-100046

[76] Chaparro, J. M., Sheflin, A. M., Manter, D. K. & Vivanco, J. M. Manipulating the soil microbiome to increase soil health and plant fertility. *Biol. Fertil. Soils* 48, 489–499. 10.1007/s00374-012-0691-4 (2012).

[77] Moreki, J. & Chiripasi, S. Poultry waste management in Botswana: A review. *Online J. Anim. Feed Res.* **1**, 285–292 (2011).

[78] Manzeke, G. M. et al. Soil fertility management effects on maize productivity and grain zinc content in smallholder farming systems of Zimbabwe. *Plant and Soil* 361, 57–69. 10.1007/s11104-012-1332-2 (2012).

[79] Chikopela, L., Kalinda, T. H., Ng’ombe, J. N. & Kuntashula, E. Cultivating sustainability: Adoption and intensity of soil fertility management technologies among rural farms in Zambia. *World Dev. Sustain.* **5**, 100174. 10.1016/j.wds.2024.100174 (2024).

[80] Abeywardana, N., Schütt, B., Wagalawatta, T. & Bebermeier, W. Indigenous agricultural systems in the dry zone of Sri Lanka: Management transformation assessment and sustainability. *Sustainability* **11**, 910. 10.3390/su11030910 (2019).

[81] Verma, J. & Verma, R. Organic fertilizers and their Impact on agricultural production system. in *Organic Fertilizers: Types, Production and Environmental Impact* (ed. Singh, R. P.), 218–232 (Science Publishers, New York, 2012).

[82] Akinloye, K., Akinloye, F., Orimoogunje, O. & Adeleke, B. Indigenous system of soil fertility management in a typical farm settlement in Osun State, Southwestern Nigeria. *Int. J. Geogr. Environ. Earth Sci.* **24**, 51–60. 10.97341/JGEESI/2020/v24i430218 (2020).

[83] Maru, Y., Gebrekirstos, A. & Haile, G. Farmers’ indigenous knowledge of tree conservation and acidic soil amendments: the role of Baabbo and Mona systems: lessons from Gedeo community, Southern Ethiopia. *Cogent Food Agric.* **5**, 1645259. 10.1080/23311932.2019.1645259 (2019).

[84] Sharma, I. P., Kanta, C., Dwivedi, T. & Rani, R. Indigenous agricultural practices: A supreme key to maintaining biodiversity. in *Microbiological Advancements for Higher Altitude Agro-Ecosystems & Sustainability* (ed. Sharma A. K.) 91–112 (Springer Nature Singapore, Singapore, 2020).

[85] Rankoana, S. A. Perceptions of climate change and the potential for adaptation in a rural community in Limpopo Province, South Africa. *Sustainability* **8**, 672. 10.3390/su8080672 (2016).

[86] Nasar, J., Alam, A., Nasar, A. & Khan, M. Z. Intercropping induce changes in above and below ground plant compartments in mixed cropping system. *Biomedical J. Sci. Tech. Res.* **17**, 13043–13050. 10.26717/BJSTR.2019.17.003054 (2019).

[87] Tripathy, S., Meena, S., Dhar, S., Paul, S. & Singh, S. Effect of row ratios and organic nutrient management on productivity and economics of Indian mustard (*Brassica juncea*) + chickpea (*Cicer arietinum*) intercropping system. *Indian J. Agricultural Sci.* **93**, 1067–1072. 10.56093/ijas.v93i10.140083 (2023).

[88] Bowles, T. M. et al. Long-term evidence shows that crop-rotation diversification increases agricultural resilience to adverse growing conditions in North America. *One Earth* **2**, 284–293 (2020).

[89] Shah, K. K. et al. Diversified crop rotation: an approach for sustainable agriculture production. *Adv. Agric.* **2021**, 8924087. 10.1155/2021/8924087 (2021).

[90] Yu, T. et al. Benefits of crop rotation on climate resilience and its prospects in China. *Agronomy* **12**, 436. 10.3390/agronomy12020436 (2022).

[91] Malapane, O. L., Musakwa, W. & Chanza, N. Indigenous agricultural practices employed by the Vhavenda community in the Musina local municipality to promote sustainable environmental management. *Heliyon* **10** (24), e33713 (2024). 39040358 10.1016/j.heliyon.2024.e33713 PMC11261858

[92] Akinola, R., Pereira, L. M., Mabhaudhi, T., De Bruin, F. M. & Rusch, L. A review of Indigenous food crops in Africa and the implications for more sustainable and healthy food systems. *Sustainability* **12**, 3493. 10.3390/su12083493 (2020). 33520291 10.3390/su12083493 PMC7116648

[93] Eliazer Nelson, A. R. L., Ravichandran, K. & Antony, U. The impact of the green revolution on indigenous crops of India. *J. Ethnic Foods* **6**, 8. 10.1186/s42779-019-0011-9 (2019).

[94] Ramya, V. & Patel, P. Health benefits of vegetables. *Int. J. Chem. Stud.* **7**, 82–87 (2019).

[95] Yahia, E. M., Carrillo-López, A., Malda, B. G., Suzán-Azpiri, H. & Quiroz, M. Q. Phytosynthesis. in *Postharvest Physiology and Biochemistry of Fruits and Vegetables* (eds Yahia, E. M. & Carrillo-Lopez, A.) 19–45 (Elsevier, 2019).

[96] Omotayo, A. O. & Aremu, A. O. How does the decision to cultivate underutilized crops influence food and nutrition security in rural areas? *J. Agric. Food Res.* **18**, 101400. 10.1016/j.jafr.2024.101400 (2024).

[97] Somasundaram, J. et al. No-till farming and conservation agriculture in South Asia–issues, challenges, prospects and benefits. *Crit. Rev. Plant Sci.* **39**, 236–279. 10.1080/07352689.2020.1782069 (2020).

[98] Mwinga, J. L., Otang-Mbeng, W., Kubheka, B. P. & Aremu, A. O. Ethnobotanical survey of plants used by subsistence farmers in mitigating cabbage and spinach diseases in OR Tambo municipality, South Africa. *Plants* **11**, 3215 (2022). 36501255 10.3390/plants11233215 PMC9741191

[99] Van Wyk, B. E. The potential of South African plants in the development of new medicinal products. *South. Afr. J. Bot.* **77**, 812–829. 10.1016/j.sajb.2011.08.011 (2011).

[100] Cheikhyoussef, A. & Embashu, W. Ethnobotanical knowledge on indigenous fruits in Ohangwena and Oshikoto regions in Northern Namibia. *J. Ethnobiol. Ethnomed.* **9**, 34. 10.1186/1746-4269-9-34 (2013). 23697554 10.1186/1746-4269-9-34 PMC3682899

[101] Maroyi, A. The gathering and consumption of wild edible plants in Nhema communal area, Midlands Province, Zimbabwe. *Ecol. Food Nutr.* **50**, 506–525. 10.1080/03670244.2011.620879 (2011). 22077930 10.1080/03670244.2011.620879

[102] Dejene, T., Agamy, M. S., Agúndez, D. & Martin-Pinto, P. Ethnobotanical survey of wild edible fruit tree species in lowland areas of Ethiopia. *Forests* **11**, 177. 10.3390/f11020177 (2020).

[103] Agarwal, R. & Chandra, V. Diversity of wild edible plants in the Mandal-Chopta forest, Uttarakhand. *J. Med. Plants Stud.* **7**, 89–92 (2019).

[104] Sasi, R. & Rajendran, A. Diversity of wild fruits in Nilgiri hills of the Southern Western Ghats-ethnobotanical aspects. *Int. J. Appl. Biology Pharm. Technol.* **3**, 82–87 (2012).

[105] Sõukand, R. & Kalle, R. Perceiving the biodiversity of food at chest-height: Use of the fleshy fruits of wild trees and shrubs in Saaremaa, Estonia. *Hum. Ecol.* 44, 265–272. 10.1007/s10745-016-9818-9 (2016).

[106] Suwardi, A. B., Navia, Z. I., Harmawan, T., Syamsuardi, S. & Mukhtar, E. Wild edible fruits generate substantial income for local people of the Gunung Leuser National park, Aceh Tamiang region. *Ethnobotany Res. Appl.* **20**, 11 (2020).

[107] Selogatwe, K. M., Asong, J. A., Struwig, M., Ndou, R. V. & Aremu, A. O. A review of ethnoveterinary knowledge, biological activities and secondary metabolites of medicinal woody plants used for managing animal health in South Africa. *Vet. Sci.* **8**, 228 (2021). 34679058 10.3390/vetsci8100228 PMC8537377

[108] Savary, S. et al. The global burden of pathogens and pests on major food crops. *Nat. Ecol. Evol.* **3**, 430–439 (2019). 30718852 10.1038/s41559-018-0793-y

[109] Laizer, H. C., Chacha, M. N. & Ndakidemi, P. A. Farmers’ knowledge, perceptions and practices in managing weeds and insect pests of common bean in Northern Tanzania. *Sustainability* **11**, 4076. 10.3390/su11154076 (2019).

[110] Dar, G. H., Bhat, R. A., Mehmood, M. A. & Hakeem, K. R. *Microbiota and Biofertilizers*, Vol. 2 (Springer, Charm, Switzerland, 2021).

[111] Mlanjeni, N. L. Identification and documentation of ethnobiological methods used by rural farmers to control stalk borers on maize in the Eastern Cape Province of South Africa. Master of Science, Department of Botany, Faculty of Science and Agriculture. (University of Fort Hare, Alice, South Africa, 2014).

[112] Skenjana, N. J. & Afolayan, A. J. A documentation of plants used by rural small-scale farmers to control maize pests in the Eastern Cape Province of South Africa. *Afr. J. Food Agric. Nutr. Dev.* **21**, 17643–17655 (2021).

[113] Skenjana, N. L. & Poswal, M. A. A survey of plants used by rural small-scale farmers to control pests of cabbage in the Eastern Cape Province, South Africa. *J. Med. Plants Economic Dev.* **2**, a57 (2018).

