# Supplementary material for: Indigenous farming methods and crop management practices used by local farmers in Madibeng local municipality, South Africa
Source: Sci Rep. 2025 Mar 14;15:8918. doi: 10.1038/s41598-025-91210-w (PMC11909181; doi:10.1038/s41598-025-91210-w)
Supplement: Supplementary file 1 — Supplementary Material 1. [file 41598_2025_91210_MOESM1_ESM.docx]

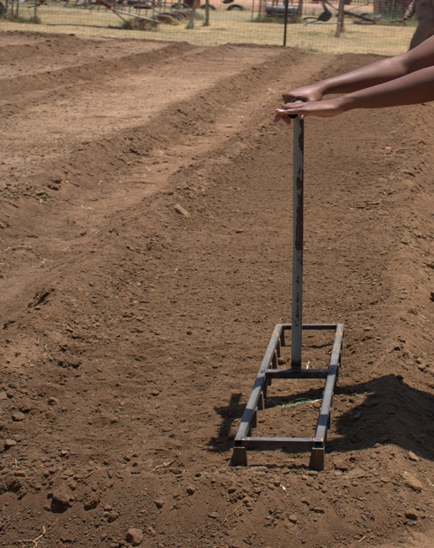


**Supplementary Figure S1:** Image of the tool used by 31% of the participants of this study for digging the seed holes
